# Eco-Friendly Formulated Zinc Oxide Nanoparticles: Induction of Cell Cycle Arrest and Apoptosis in the MCF-7 Cancer Cell Line

**DOI:** 10.3390/genes8100281

**Published:** 2017-10-20

**Authors:** Amin Boroumand Moghaddam, Mona Moniri, Susan Azizi, Raha Abdul Rahim, Arbakariya Bin Ariff, Mohammad Navaderi, Rosfarizan Mohamad

**Affiliations:** 1 Department of Bioprocess Technology, Faculty of Biotechnology and Biomolecular Sciences, Universiti Putra Malaysia, 43400 UPM Serdang, Selangor, Malaysia; amin.broomandm@yahoo.com (A.B.M.); mona_moniri6@yahoo.com (M.M.); arbarif@upm.edu.my (A.B.A.); 2 Young Research and Elite Club, Sabzevar Branch, Islamic Azad University, Sabzevar, Iran; 3 Department of Cell and Molecular Biology, Faculty of Biotechnology and Biomolecular Sciences, Universiti Putra Malaysia, 43400 UPM Serdang, Selangor, Malaysia; raha@upm.edu.my; 4 Bioprocessing and Biomanufacturing Research Centre, Faculty of Biotechnology and Biomolecular Sciences, Universiti Putra Malaysia, 43400 UPM Serdang, Selangor, Malaysia; 5 Young Research and Elite Club, Parand Branch, Islamic Azad University, Parand, Iran; navaderimohammad@yahoo.com; 6 Institute of Tropical Forestry and Forest Products, Universiti Putra Malaysia, 43400 UPM Serdang, Selangor, Malaysia

**Keywords:** ZnO NPs, annexin V, cell cycle, apoptosis, *Pichia kudriavzevii*, green method

## Abstract

Green products have strong potential in the discovery and development of unique drugs. Zinc oxide nanoparticles (ZnO NPs) have been observed to have powerful cytotoxicity against cells that cause breast cancer. The present study aims to examine the cell cycle profile, status of cell death, and pathways of apoptosis in breast cancer cells (MCF-7) treated with biosynthesized ZnO NPs. The anti-proliferative activity of ZnO NPs was determined using MTT assay. Cell cycle analysis and the mode of cell death were evaluated using a flow cytometry instrument. Quantitative real-time-PCR (qRT-PCR) was employed to investigate the expression of apoptosis in MCF-7 cells. ZnO NPs were cytotoxic to the MCF-7 cells in a dose-dependent manner. The 50% growth inhibition concentration (IC_50_) of ZnO NPs at 24 h was 121 µg/mL. Cell cycle analysis revealed that ZnO NPs induced sub-G_1_ phase (apoptosis), with values of 1.87% at 0 μg/mL (control), 71.49% at IC_25_, 98.91% at IC_50_, and 99.44% at IC_75_. Annexin V/propidium iodide (PI) flow cytometry analysis confirmed that ZnO NPs induce apoptosis in MCF-7 cells. The pro-apoptotic genes *p53*, *p21*, *Bax*, and *JNK* were upregulated, whereas anti-apoptotic genes *Bcl-2*, *AKT1*, and *ERK1/2* were downregulated in a dose-dependent manner. The arrest and apoptosis of MCF-7 cells were induced by ZnO NPs through several signalling pathways.

## 1. Introduction

Breast cancer is one of the most frequent types of cancers in women, and overall it is the second most common form of cancer worldwide. As many as 25% of cases of cancer (1.67 million cases) and 15% of deaths in women (522,000 individuals) were caused by forms of breast cancer, as reported by GLOBOCAN [1].

The balance lost between the proliferated cells and apoptosis is a hallmark that intensifies the failure of damaged cells to be wiped out via apoptosis. Activating apoptotic paths in cells affected by tumour is a crucial approach to cancer therapy. Several natural products that are considered potentially powerful sources of anticancer drugs apply anti-tumour effects by inducing apoptosis [2]. Extracellular or intracellular signals activate apoptosis, which in turn provokes numerous signals of vague contraction or DNA fragmentation [3]. Additionally, the deregulations that cause the initiation and development of cancers represent hundreds of genes or signalling cascades [4]. Furthermore, numerous genes like *p53*, *p21*, *JNK*, *Bax*, *Bcl-2*, *AKT*, and *ERK1/2* engage in apoptotic pathways. Induced by gene alterations engaging in mitosis and chromosome separation, *p53* is essential for cellular aging [5]. To keep the genome stable, cell cycle checkpoints, DNA repair, and apoptosis can be activated by *p53* [3]. The progress of the majority of malignancies is highly dependent on the change or loss of *p53* [6]. The transcription of *Bcl-2* family members, particularly *Bcl-2* and *Bax*, can also be controlled by *p53*. In addition, cyclin-dependent kinase inhibitors or in other words, the transcription of *p21*, can be activated by *p53* when DNA is damaged, which can affect the development of the cell cycle through an interaction with various transcription factors leading to apoptosis [7,8]. The overexpression of anti-apoptosis *Bcl-2* has been involved in various carcinomas [9].

The Jun N-terminal kinase (*JNK*) pathway plays an important role in apoptosis. Apoptosis can be triggered by the regulation of pro-apoptotic genes via different types of transcription factor transactivation signalling or through manipulation proteins of pro- and anti-apoptotic proteins in mitochondria [10]. Unlike the *JNK* pathway, the pathway of extracellular signal-regulated kinase (*ERK*) is relevant to the proliferation, survival, and differentiation of cells [11]. Meanwhile, cell survival, cell growth, and angiogenesis are regulated by the *PI3-K/AKT* pathway [12]. Apoptosis in cancerous cells is elicited by inhibition of the *AKT* and *ERK* pathway [13].

Chemotherapy is one of the main systemic treatments for early breast cancer, and its use has led to an improvement in the survival of women diagnosed with breast cancer. However, the non-specific systemic delivery, causing damage to normal, unaffected tissue, is a major problem of chemotherapy drugs [14]. Nanomedicine has recently emerged as a better choice for treating some common cancers, resulting in many nanoparticles being used as treatment in cancer cell lines. Zinc oxide (ZnO) shows biocompatibility compared to different materials [15]. ZnO is an inorganic compound listed as “Generally Recognized as Safe” (GRAS) by the USA Food and Drug Administration (FDA) (21CFR182.8991) [16]. Although Zn is a necessary trace element involved in some biological processes [17,18], when its local concentration increases, as has been shown, it will kill cells [19]. It has been reported that Zn^2+^ considerably affects cancer cells, indicating gene expression reduction and apoptosis induction [20]. The induction of apoptosis by zinc in cancers appears to be cell type-specific [20]. Zn apoptosis effects indicate that ZnO nanostructures can be utilized as an agent for anticancer, providing a possible target for the development of anti-tumour agents [20]. What is more surprising in vitro observations is that cancer cells that are dramatically less toxic to normal cells can be preferentially killed by ZnO NPs [21].

The technique for synthesizing ZnO nano-sized materials is a challenge for attaining standard antitumour therapy. Our previous study investigated the antioxidant and antibacterial activity of biosynthesized ZnO NPs using a new strain of yeast (*Pichia kudriavzevii* GY1) [22]. In this study, ZnO NPs were examined to evaluate breast cancer cell (MCF-7) anti-proliferation activity. To our knowledge, no previous studies have reported the potential use of ZnO nanoparticles by *P. kudriavzevii* in breast cancer treatment (in the MCF-7 cell line). As a result, a better insight into the anticancer activities of ZnO NPs and the cytotoxic effects of their constituents can contribute to facilitating the improvement of auspicious cancer therapeutics for use in nanomedicine.

## 2. Materials and Methods

### 2.1. Cell Culture

The human breast cancer cell line (MCF-7) utilized in this study was obtained from the American Culture Collection (ATCC, Rockville, MD, USA). Phenol-red-free Roswell Park Memorial Institute medium (RPMI 1640) with L-glutamine (Sigma-Aldrich, Steinheim, Germany), supplemented with 10% foetal bovine serum (FBS) (PAA, Pasching, Austria) and 1% penicillin–streptomycin (PAA, Pasching, Austria) were used to culture cells. Each experiment used cells with a passage number less than 20. All cells were maintained at 37 °C in a humidified incubator containing 5% CO_2_.

### 2.2. In Vitro Cytotoxicity Assay

In vitro cytotoxicity study was determined using MTT assay [23]. Concisely, 96 well plates were used to seed cells at a density of 1 × 10^6^ cells per 100 µL. Different concentrations of ZnO NPs (0, 62.5, 125, 250, 500, and 1000 μg/mL) were applied to treat cells after incubation at 37 °C for a period of a whole day. In addition, the cytotoxicity assay of tamoxifen was investigated in various concentrations (0, 6.25, 12.5, 25, 50, and 100 μg/mL) [24]. A volume of 10 µL of MTT solution was added to each well and further incubated for 24 h in relative humidity at 37 °C with 5% CO_2_. RPMI-1640 media without any samples was used as a negative control. Tamoxifen is a breast cancer treatment drug that was used as a positive control for the MCF-7 cell line. Each concentration of ZnO NPs was assayed in triplicate. The percent cell viability was calculated by following equation [25]:

$$\mathrm{Cell}\;\mathrm{viability}\;\left(\%\right)=\frac{\mathrm{OD}\;\mathrm{of}\;\mathrm{Control}-\mathrm{OD}\;\mathrm{of}\;\mathrm{ZnO}-\mathrm{NP}\;\mathrm{treatment}}{\mathrm{OD}\;\mathrm{of}\;\mathrm{Control}}\;\times \;100$$

The 50% growth inhibition concentration (IC_50_) was calculated from a plotted dose–response curve.

### 2.3. Cell Cycle Analysis by Flow Cytometry

Culture flask cells measuring 25 cm^2^ were used to seed the cultured cells at a density of 1 × 10^6^ per mL, allowing the cell to adhere to the flask wall overnight. Treated cells had concentrations of ZnO NPs capable of growth inhibition of 25, 50, and 75% (IC_25_, IC_50_, and IC_75_, respectively) within 24 h. At 1000 rpm for 10 min, the treated and untreated (negative control) cells were collected and centrifuged. The supernatant was disposed of and cold phosphate buffered saline (PBS) was used to wash the pellet. Cells were re-suspended in 500 µL PBS and subsequently fixed in 70% cold ethanol for at least 2 h at −20 °C. Cells were centrifuged at 1000 rpm for 5 min, the supernatant was discarded, and the cells were washed with PBS. The supernatant was disposed of and the cells were incubated with a mixture of 500 μL PI/RNase (400 µL propidium iodide and 100 µL ribonuclease A). Before being analysed, stained cells were incubated in darkness for a period of 30 min at room temperature. BD LSRFortessa™ Cell Analyzer (Becton Dickinson, San Diego, NJ, USA) determined the cell cycle profile for 10,000 events per sample. Analytical software BD FACSDavia™ data was presented by percentage of cells compared to the populations of the untreated control.

### 2.4. Apoptosis Detection by Annexin V/Propidium Iodide Assay

Referring to the manufacturer’s protocol, Annexin V-FITC (Fluorescein isothiocyanate) was performed to determine cell death mode. The cells were seeded in 25 cm^2^ culture flasks and incubated at 37 °C in a humidified, 5% CO_2_ incubator atmosphere for 24 h to allow them to attach. Culture medium was discarded and cells were treated with ZnO NPs at IC_25_, IC_50_, and IC_75_ for 24 h. After incubation, detached and adherent cells were collected by combining the spent medium and trypsin-EDTA-treated cells. Harvesting of treated and untreated (negative control) cells was done by centrifugation (1000 rpm for 5 min) and then they were washed twice using cold PBS. After the supernatant was discarded, the pellet was re-suspended in 1× binding buffer. Then, 5 μL of annexin V-FITC and 10 μL of propidium iodide were added to each suspension in order to stain the cells. Suspensions were then gently vortexed and incubated while in darkness at room temperature for 10 min. BD LSRFortessa™ Cell Analyzer (Becton Dickinson) measured the cell death induction.

### 2.5. Gene Expression by quantitative real-time-PCR

RNA extraction of untreated (negative control) and treated MCF-7 cells with different concentrations (IC_25_, IC_50_, and IC_75_) of ZnO NPs was performed through using an RNeasy Mini kit (Qiagen, Inc., Valencia, CA, USA). The procedure was performed as per the manufacturer’s instructions. These instructions were also used as the template for quantitative real-time-PCR (qRT-PCR) to synthesize cDNA from purified RNA with an RT^2^ First Strand Kit (Qiagen). To perform qRT-PCR, the Corbett Rotor-Gene 6000 (Qiagen) was utilized. Finally, a pre-mix with a final volume of 25 μL was prepared, consisting of 12.5 μL of RT^2^ SYBR^®^Green ROX ^TM^ FAST mastermix (Qiagen), 1 μL of primers (RT^2^ qPCR Primer Assays, Qiagen), 1 μL of cDNA, and 10.5 μL RNase-free water. Table 1 shows the primer pairs of target genes and GAPDH that were chosen from the Primer Bank website (www.ncbi.nlm.nih.gov).

The conditions of default PCR were as follows: the PCR conditions were set to 95 °C to activate the enzyme for 10 min, and then 40 cycles for 15 s at 95 °C (denaturation) were performed, followed by a 30-s cycle at 60 °C (annealing and synthesis). Finally, to check and justify the results, the dissociation curve was built right after the PCR run. The expression of all genes (*p53*, *p21*, *Bax*, *JNK*, *Bcl-2*, *AKT1*, and *ERK1/2*) was compared with the housekeeping gene (GAPDH) using 2^−ΔΔCt^ approach which is the result of the equation: 2 ^ΔΔCt^ = 2^Ct (treated cells)−Ct(control cells)^, where 2 is a derivation of the amplification efficiency doubling the template in each cycle while ascending in strength.

### 2.6. Statistical Analysis

All data were analysed using Graph pad prism software version 7.01 (GraphPad Software Inc., La Jolla, CA, USA). At the level of *p* ≤ 0.05, one-way analysis of variance (ANOVA) and differences were found to be significant.

## 3. Results

### 3.1. MTT Assay

**Figure 1:**
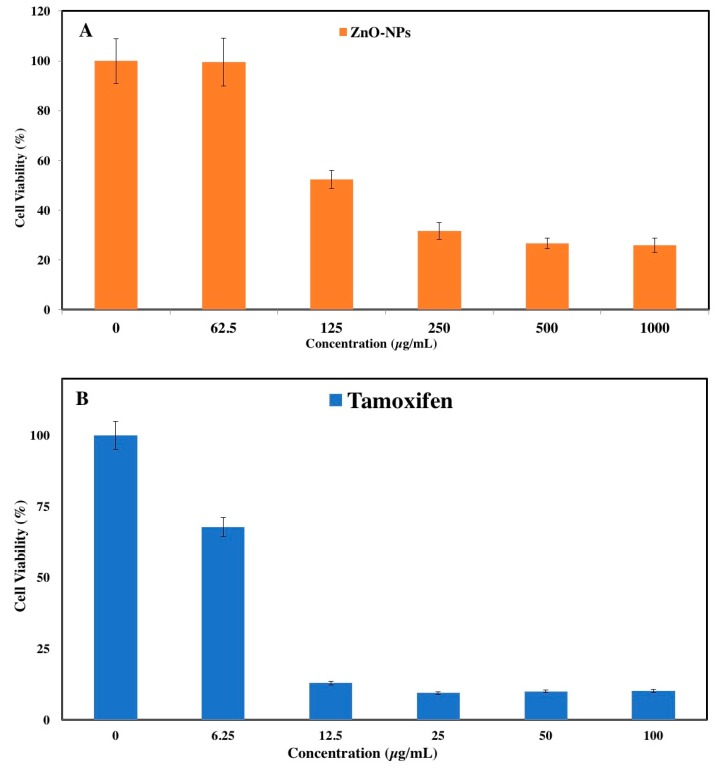
(**A**) Anti-proliferative activity of zinc oxide nanoparticles (ZnO NPs) with respect to MCF-7; (**B**) Effect of tamoxifen on MCF-7 cells.

The anticancer activity of ZnO NPs was investigated using the MTT assay on a human breast cancer cell line (MCF-7). MTT solution was mainly applied to stablish the applicability of cells, and it leads to mitochondrial dysfunction [23]. The ZnO NPs exhibited dose-dependent inhibition against the proliferation of cancer cell lines in the present study. After 24 h of treatment, the IC_50_ value was 121 µg/mL for MCF-7 cells (Figure 1A). Moreover, untreated cells were used as a negative control. Based on previous study, the ZnO NPs showed a selective anticancer effect, with no cytotoxicity in normal cells (Vero cell), and an IC_50_ greater than 100 µg/mL [22]. In addition, in reference to another study reported in the literature, it has recently been demonstrated that ZnO NPs induce the death of cancerous cells while having no cytotoxic effect on normal cells [26]. Other research also mentioned that ZnO NPs induce cell death in breast and prostate cancer cell lines, but have no major cytotoxic effects on normal breast and prostate cells [27]. Nevertheless, the IC_50_ of tamoxifen (a commercial chemotherapy drug) was used against MCF-7 cell line as a positive control, which showed that cytotoxicity in cancer cells is capable of being induced (Figure 1B). After 24 h of incubation the IC_50_ value for the MCF-7 cell line was 8 µg/mL.

### 3.2. The Effect of Cell Cycle Distribution

**Figure 2:**
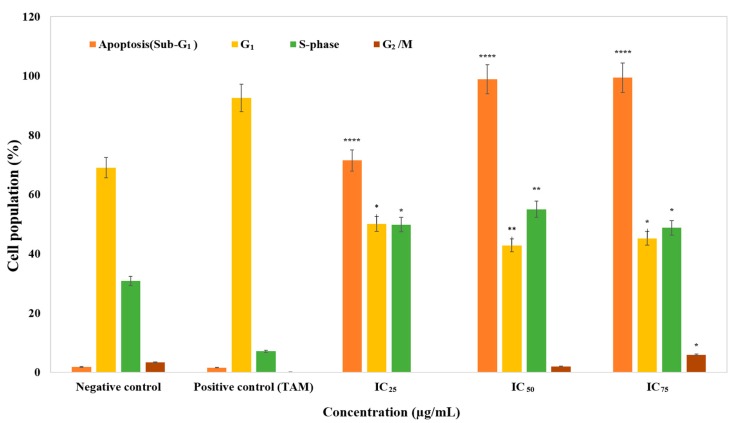
The cell cycles of MCF-7 cells treated with ZnO NPs. Cell populations are presented for each phase of the cell cycle for negative control (untreated cell), positive control (tamoxifen), and cells exposed to different concentrations of ZnO NPs for 24 h. Data represent the mean ± standard deviation (SD) (*n* = 3). More information in shown in Appendix A. * *p* ≤ 0.0.5; ** *p* ≤ 0.0; *** *p* ≤ 0.001; **** *p* ≤ 0.0001. TAM: Tamoxifen.

Through measuring the distribution of cell cycle, proliferation prevention was tested. After the MCF-7 cells were treated with ZnO NPs at different concentrations (IC_25_, IC_50_, and IC_75_) for 24 h, they were collected, fixed, and stained with propidium iodide, and the cell populations of each phase were determined via a flow cytometer. For cells treated with apoptosis-inducing agents, a subpopulation of cells appeared before the G_1_ peak, which is called the sub-G_1_ (apoptosis) peak. It is believed that this population with lesser stability is the outcome of endonuclease activation and subsequent DNA leakage of the cells. Since the immediate reduction in DNA content is not shown by necrotic cells, the difference between apoptotic and necrotic cells will be obvious. The degree or progression of apoptosis in a cell population can be measured by quantifying the ratio of cells in the sub-G_1_ in relation to the other phases [28]. As Figure 2 shows, the population of sub-G_1_ (indicating apoptotic cells) grew in a dose-dependent manner from 1.87% at 0 μg/mL (control) to 71.49% at IC_25_, 98.91% at IC_50_, and 99.44% at IC_75_, after exposure to ZnO NPs for 24 h (*p* < 0.0001). While the other portion of non-apoptotic cells exhibited no major changes, the population of G_1_ declined as the population of sub-G_1_ cells increased. When compared with the positive and negative control, the cell population of sub-G_1_ was shown to have an outstanding increase (Figure 2). On the other hand, in the S-phase it can be seen that the percentage of cells was increased compared to the control, in other words, the cells arrested. Deregulation of the cell cycle checkpoints could cause abnormal cell proliferation and cancer development [29]. Progression through the S-phase is controlled by the monitoring of replication checkpoints and moderation of DNA synthesis. In the event of DNA damage, this checkpoint prevents cell-cycle progression [30]. Hence, it was possible that the increment of cells in S-phase was due to the incorporation of NPs into damaged DNA during the process of DNA replication. On the other hand, tamoxifen is a selective oestrogen-receptor modulator [31] that has been used to treat both early and advanced breast cancer for more than three decades [32]. It has been thoroughly evaluated for the reduction of the risk of both invasive and non-invasive breast cancer in women at increased risk [33,34,35]. In this research, as a common commercial chemotherapy drug, tamoxifen was used as a positive control against the MCF-7 cell line. The MCF-7 cell is the most commonly used breast cancer cell line in the world, and was established in 1973 at the Michigan Cancer Foundation [36]. The popularity of MCF-7 is largely due to its exquisite hormone sensitivity through expression of the oestrogen receptor (ER), making it an ideal model to study hormone response [37]. According to tamoxifen results, it can be seen that the percentage of cells in G_1_ phase was increased and the number of cells in S-phase declined dramatically compared to the negative control. In other words, tamoxifen led to G_1_ arrest and a decline in proliferation. One useful approach in cancer therapy is the induction of apoptosis. Many cellular molecular biological aspects like DNA fragmentation, cell shrinkage, and caspase cascade activation are shown in apoptotic cells [38]. Overall, all these results suggested that ZnO NP-induced growth inhibitory effect is associated with the occurrence of apoptosis, more than cell cycle arrest. Thus, the apoptotic cell death of ZnO NPs was further analysed.

### 3.3. ZnO Nanoparticles Induced Apoptosis in MCF-7 Cells

**Figure 3:**
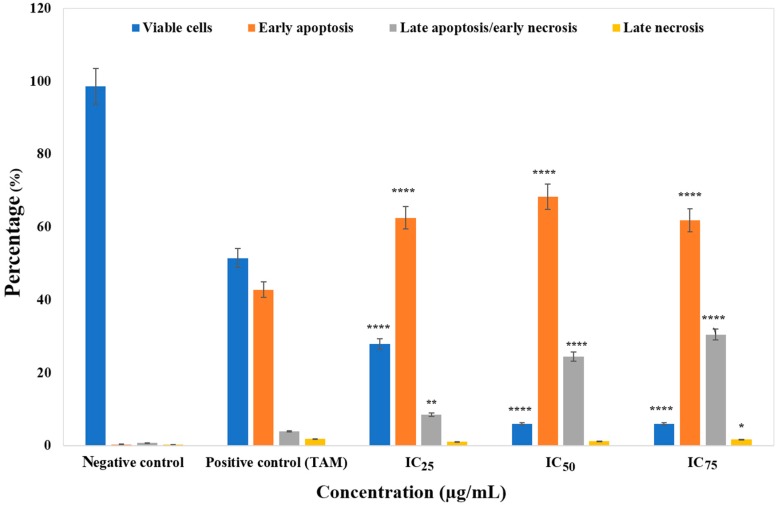
The percentage of viable, early apoptotic, late apoptotic/early necrotic, and late necrotic cells with respect to negative control (untreated), positive control (tamoxifen), and MCF-7 cells treated for 24 h as determined by a flow cytometer instrument. Cells were exposed to different concentrations of ZnO NPs for 24 h. Data represent the mean ± SD (*n* = 3). More information on cell percentages is provided in Appendix B. * *p* ≤ 0.0.5; ** *p* ≤ 0.0; *** *p* ≤ 0.001; **** *p* ≤ 0.0001.

Flow cytometry was applied to quantitatively detect apoptosis rates through annexin V-FITC/PI staining. MCF-7 cell treatment with different concentrations (IC_25_, IC_50_ and IC_75_) of ZnO NPs was carried out for 24 h. Stained cells can be distinguished into four groups, namely viable (annexin V^−^ PI^−^), early apoptotic (annexin V^+^ PI^−^), late apoptotic (annexin V^+^ PI^+^) and necrotic (annexin V^−^ PI^+^) cells, using a flow cytometer instrument. As illustrated in Figure 3, 98.60% of negative control MCF-7 culture cells were viable. No apoptosis occurred after 24 h of incubation. Treating cells with ZnO NPs, the viable cell percentage reduced significantly at IC_25_, IC_50_, and IC_75_ (27.94 ± 5.69%, 5.98 ± 0.73% and 5.99 ± 0.29%) respectively (Figure 3). Furthermore, the percentage of cells at an early apoptotic stage increased significantly at all concentrations (IC_25_ (62.53 ± 6.02%), IC_50_ (68.32 ± 2.10%) and IC_75_ (61.85 ± 3.68%)) compared to the negative control (0.36 ± 0.54%) (*p* < 0.0001). Figure 3 shows that the greater the increase in dosage, the greater the population of late apoptotic cells (8.51 ± 0.31%, 24.47 ± 1.18%, 30.50 ± 3.07%).

### 3.4. Gene Expression Analysis of Apoptosis

**Figure 4:**
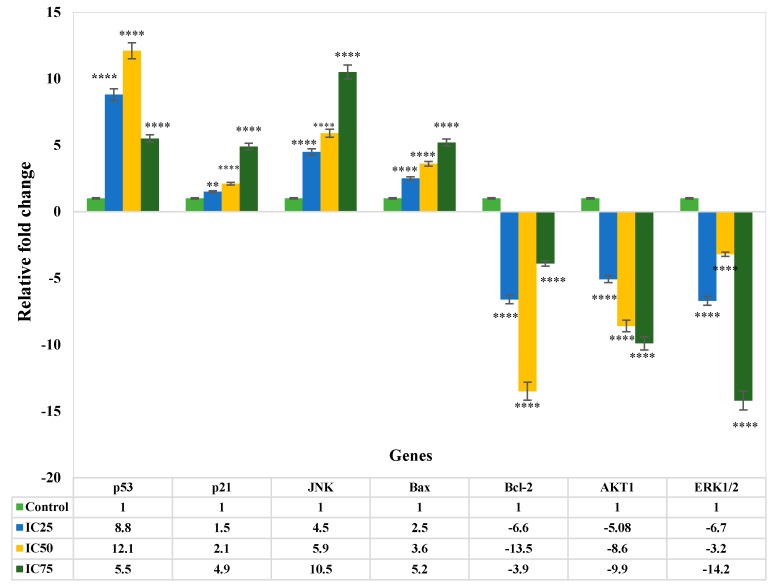
Gene expression levels in MCF-7 cells following treatment with ZnO Ps. Relative fold change of gene expression in ZnO NP-treated cells at 24 h, in comparison to the control. The values represent the mean ± STD (*n* = 3). * *p* ≤ 0.0.5; ** *p* ≤ 0.0; *** *p* ≤ 0.001; **** *p* ≤ 0.0001.

In this study, qRT-PCR was utilised to analyse the mRNA levels of apoptotic markers such as *Bcl-2*, *Bax*, *p53*, *p21*, *AKT1*, *ERK1/2*, and *JNK* in MCF-7 cells in the exposure of ZnO NPs at different concentrations (IC_25_, IC_50_, and IC_75_) for 24 h (Figure 4). The expression of the *p53* gene in the treated MCF-7 cells was shown to be upregulated by 8.8 and 12.1-fold using IC_25_ and IC_50_ (*p* < 0.0001). The *p21* expression was notably upregulated (*p* < 0.0001) at 24 h by approximately 1.5, 2.1, and 4.9-fold, at the tested concentrations of IC_25_, IC_50_, and IC_75_, respectively. Moreover, the expression level of total *JNK* increased significantly (IC_25_: 4.5-fold, IC_50_: 5.9-fold, and IC_75_: 10.5-fold) compared to the control in 24 h (*p* < 0.0001). (Figure 4). A significant increase in mRNA level of *Bax* was seen in MCF-7 cells after treatment with IC_50_ and IC_75_ (3.6 and 5.2-fold) respectively (*p* < 0.0001). On the other hand, treated MCF-7 cells showed Bcl-2 downregulation by ZnO NPs at IC_25_ and IC_50_, by –6.6 and –13.5 fold, respectively. Similarly, ZnO NPs had a major effect on the expression of *AKT1* in MCF-7 cells, since it decreased the levels of *AKT1* with IC_25_, IC_50_, and IC_75_ concentrations by –5.08, –8.6 and –9.9 fold, respectively. Figure 4 shows that the expression of *ERK1/2* of MCF-7 cells treated with IC_25_, IC_50_, and IC_75_ of ZnO NPs was substantially downregulated (*p* < 0.0001) at 24 h by –6.7-, –3.2, and –14.2 fold, respectively, compared to control.

## 4. Discussion

In the present study, the green synthesis of ZnO NPs showed strong cytotoxic characteristics against MCF-7 cells (Figure 1). The cell viability of breast cancer cells noticeably decreases with an increase in ZnO NP concentration. There are various parameters that can be considered to study the cytotoxic effect of nanoparticles, such as shape, size, surface charge, and dissolution of NPs [39]. Moreover, Wahab and colleagues also reported the viability of cancer cell line was reduced by ZnO NPs and that the degree of reduction was concentration/dose-dependent [40]. In another study, the investigation of the toxicity effect of ZnO NPs using the green approach was compared with the ZnO NP commercial type. They reported that the cytotoxicity effect of green synthesis was much better compared to commercial type against the MCF-7 cell line [41]. In addition, Sivaraj et al. reported that the cytotoxicity of the ZnO NPs was evaluated against MCF-7 at various concentrations (6.5–100 μg/mL). Moreover, by increasing concentration the toxicity effect was increased [42].

The cell cycle includes interphases (G_1_, S, and G_2_) and mitosis (M). Cells increase in size, produce RNA, and synthesize proteins for DNA formation during the G_1_ period. In normal conditions, during the S phase DNA replicate and cells continue to grow; new proteins are produced at the G_2_ phase. It is at the M stage where nuclear and cytoplasmic divisions take place [43]. Moreover, one strategy in the progression of anticancer drugs is the regulation of cancer cell cycle [44]. According to the result of cell cycle analysis, determined by analysis from flow cytometry, ZnO NPs can induce apoptosis in MCF-7. Based on our result, inhibition of the cell growth with IC_50_ and IC_75_ ZnO NPs at 24 h (Figure 2) was due to induction of apoptosis at the sub-G_1_ phase. Wahab et al. reported that MCF-7 cells which were treated with ZnO NPs showed a significant induction of apoptosis. This was shown by the fact that roughly 26% of treated cells appeared in the sub-G_1_ phase of the cell cycle, as compared to 2.8% of the untreated control cells. The increase in the concentration of nanoparticles improved the degree of apoptosis [40].

The annexin V-FITC/PI flow cytometry analysis stablished the induction of apoptosis by ZnO NPs in MCF-7 cells (Figure 3). Necrosis and apoptosis are two distinct modes of cell death which differ in morphology, mechanisms, and incidence [45]. Cell detachment and shrinkage, bleb separation (protrusions of the plasma membranes) and the formation of apoptotic bodies packed densely with cellular organelles and nuclear fragments are the main initiators of apoptosis [46]. In vivo, apoptosis does not go through lysis bodies, as macrophages or adjacent cells surround them. Conversely, in vitro, due to the absence of phagocytes, the formed apoptosis bodies will finally swell and the process of apoptosis moves on to an autolytic necrotic outcome [47,48,49]. Here, the term late apoptosis or secondary necrosis, which is the natural outcome of the complete apoptotic program, is used to describe dead cells that have reached this state via apoptotic programming as named in [49,50]. In apoptosis, the cytoplasmic and the lysosomal membrane remain intact [49]. Necrosis, on the other hand, has been characterized as a passive, accidental cell death process that elicits uncontrolled inflammatory cellular responses [51]. The most significant features of necrosis are cell swelling, lysosomal membrane permeabilisation, and cell membrane permeabilisation [49]. As apoptosis is considered to be a regulated and a controlled process, its occurrence during cancer treatment has received great attention [52,53].

The possible mechanism of death of the MCF-7 cells after exposure to the ZnO NPs was further explored by annexin V/PI assay, which measures apoptosis. In apoptotic cells, the membrane phospholipid phosphatidylserine (PS) is translocated from the inner to the outer leaflet of the plasma membrane [54]. This translocation of PS to the outer leaflet of the lipid bilayer occurs very early in the apoptotic process [48]. In a calcium-dependent manner, annexin V, which is an internal protein in the human body and has a high affinity to bind to PS, can detect the PS that has been exposed. This binding of annexin V to PS can be detected using fluorescently-labelled annexin V and a flow cytometer. PI, which is used to differentiate applicable from inapplicable cells, is a standard flow cytometrically-applicable probe. PI is not included in applicable cells with intact membranes. However, the membranes of the dead or damaged cells are permeable for PI staining [55]. Therefore, staining with fluorochromes, including FITC-conjugated annexin V (FITC-annexin V) is typically used in conjunction with PI [56]. The analysis revealed that with 24-h treatment at varying concentrations, i.e., IC_25_, IC_50_, and IC_75_, ZnO NPs yielded higher percentages of early apoptotic cells, while only a low percentage of cells died via the necrosis pathway, as dying cells by apoptosis were finally degraded to necrotic ones as a result of losing their ability to repair DNA in late apoptosis. Overall, this result suggested that ZnO NPs induced the early apoptotic rather than necrotic pathway. In a similar study, Deng and colleagues also reported, via annexin V-FITC and PI assay, that ZnO NPs can induce apoptosis in mouse neural stem cells (NSCs). After a whole day of treatment with ZnO NPs, the number of apoptotic cells reached 55.6% [57].

The apoptosis induction by ZnO NPs in investigating molecular pathways was carried out utilising the qRT-PCR technique. Expression of *p53*, *p21*, *JNK*, and *Bax* was upregulated, while in contrast, *Bcl-2*, *AKT1*, and *ERK 1/2* was downregulated following treatment with ZnO NPs. The tumour suppressors *p53* and *p21* of MCF-7 cells were upregulated in the present research (Figure 4). The upregulation of *p53* by ZnO NPs has been suggested to trigger p21 protein accumulation, resulting in a sub-G_1_ phase cell cycle for apoptosis induction in MCF-7 cells [58,59,60]. *p53/p21* is involved in cell cycle control, apoptosis, and genomic stability maintenance. Upon DNA damage, *p53* starts repairing the damage by inducing cell cycle arrest at different checkpoints. Then, apoptosis kills those cells that are not repaired [59].

Involvement from members of the *MAPK* family, such as *ERK* and *JNK*, is also crucial in regulating cancer cell proliferation. The key factors in intrinsic pathways can indirectly be promoted by ZnO NPs modulating *ERK*. Throughout this study, apoptosis was constantly triggered by *ERK* inhibition [61]. The activation of *JNK*, as a stress-responsive kinase, was reported to induce apoptosis in a variety of cancer cells, including MCF-7 cells [62]. Following treatment with ZnO NPs, the *JNK* gene was upregulated by over 5-fold in this research. It can be suggested that bioactive component excitement in ZnO NPs, which in turn induces growth inhibition and apoptosis in MCF-7 cells, can activate *JNK* [63]. Hence, modulating the expression of the *Bcl-2* gene might be the mechanism through which ZnO NPs induce apoptosis. By preventing mitochondrial membrane potential loss, *Bcl-2* group members have long been claimed to have a pivotal role in cell viability maintenance. Overexpression of *Bcl-2* stops apoptosis, whereas upregulation of *Bax* induces apoptosis in cancer cells [64,65].

In our study, the mRNA expression level of *Bax* was upregulated, with downregulation of *Bcl-2* following treatment with ZnO NPs. The elevated *Bax/Bcl-2* ratio and the depolarization of mitochondrial membrane potential suggest that ZnO NPs induced apoptosis is mitochondria-dependent.

The expression of *AKT1* gene (Figure 4) was downregulated in this research, suggesting *AKT* pathway involvement in ZnO NP-induced apoptosis. The control of balance between survival and apoptosis is facilitated by a serine-threonine kinase, i.e., *AKT* [66].

One correlation study at King Saud University (Saudi Arabia) showed the effect of ZnO NPs on mRNA levels of apoptotic markers by qRT-PCR. After treatment of HepG2 cells (liver cancer cells) with ZnO NPs, mRNA levels of the cell cycle checkpoint protein *p53* and the pro-apoptotic protein *Bax* were shown to be upregulated. The mRNA expression levels of tumour suppression gene *p53* was reported to be 1.9-fold higher and their levels of pro-apoptotic gene *Bax* as well as anti-apoptotic gene *Bcl-2* were reduced in the exposed cells compared to the untreated cells [40].

In other research, the expression of pro-apoptotic (*Bax*, *Noxa* and *Puma*) and anti-apoptotic (*Bcl-xl*) genes of human epidermal keratinocytes (HaCaT) after 24-h exposure with ZnO NPs was evaluated. Based on the results, expression of the pro-apoptotic genes increased significantly, while the expression of the anti-apoptotic gene decreased. According to the findings of this research, ZnO NPs induced cell cycle arrest at G2/M, which was associated with changes in epigenetic and was accompanied by *p53-Bax* mitochondrial pathway-mediated apoptosis [67].

## 5. Conclusions

The anti-cancer effects of ZnO NPs on MCF-7 cell were shown in varying dosages. Through the inhabitation of cell proliferation of the cancer cells, the effect was mediated. The underlying mechanisms include stimulation of cell-specific G_0_/G_1_, S, and G_2_/M cell cycle arrest, and the induction of apoptosis through both extrinsic and intrinsic apoptotic pathways. In addition, the ZnO NP-induced apoptosis in MCF-7 cells is likely to reflect downregulation of *Bcl-2* and *AKT1* and *ERK1/2*, while *p21*, *p53*, *JNK* and *Bax* were upregulated. The hypothesis that green synthesis of ZnO NPs using the new strain of yeast *P. kudriavzevii* GY1 can potentially be used in certain types of anti-cancer therapy is supported by this research.

## Figures and Tables

**Table 1:** Genes used in quantitative real-time-PCR (qRT-PCR).

Genes	Forward Primer	Reverse Primer
*p-53*	5′-AGGTGACACTATAGAATA-3′	5′-GGGATATCACTCAGCATG-3′
*p-21*	5′-AGGTGACACTATAGAATA-3′	5′-GGGATATCACTCAGCATG-3′
*JNK*	5′-AGGTGACACTATAGAATA-3′	5′-GTACGACTCACTATAGGG-3′
*Bax*	5′-AGGTGACACTATAGAATA-3′	5′-GGGATATCACTCAGCATG-3′
*Bcl-2*	5′-AGGTGACACTATAGAATA-3′	5′-GGGATATCACTCAGCATG-3′
*AKT1*	5′-AGGTGACACTATAGAATA-3′	5′-GTACGACTCACTATAGGG-3′
*ERK1/2*	5′-AGGTGACACTATAGAATA-3′	5′-GTACGACTCACTATAGGG-3′
GAPDH	5′-AGGTGACACTATAGAATA-3′	5′-GTACGACTCACTATAGGG-3′

**Figure A1:**
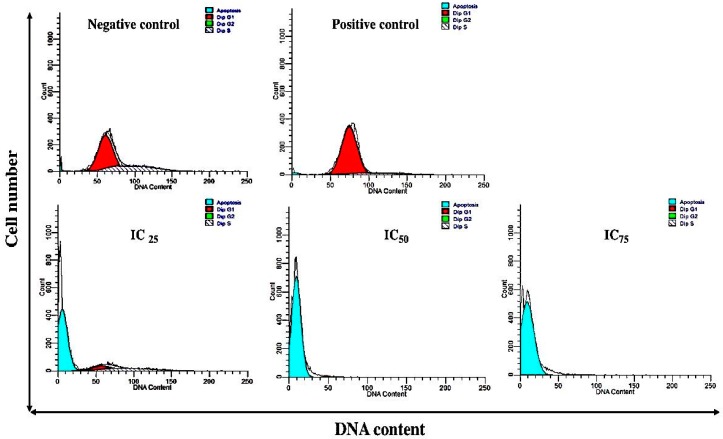
Shows the cell cycle of MCF-7 cells treated with ZnO NPs.

**Figure A2:**
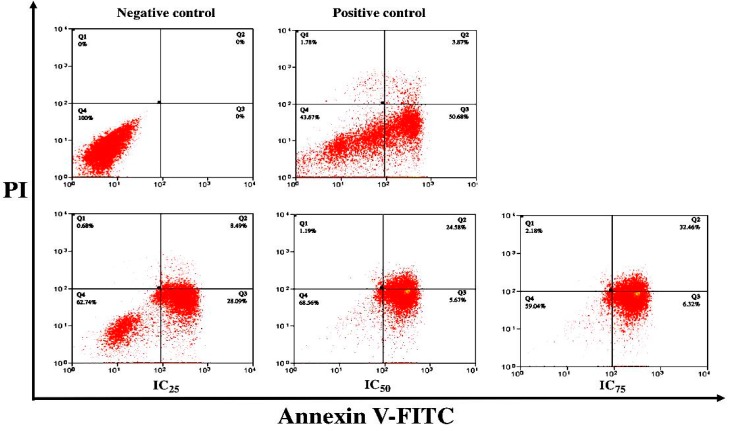
The percentage of viable, early apoptotic, late apoptotic/early necrotic, and late necrotic cells of negative control (untreated), positive control (tamoxifen) and MCF-7 treated cells for 24 h as determined by a flow cytometer instrument.
